# MDM2 facilitates adipocyte differentiation through CRTC-mediated activation of STAT3

**DOI:** 10.1038/cddis.2016.188

**Published:** 2016-06-30

**Authors:** P Hallenborg, M Siersbæk, I Barrio-Hernandez, R Nielsen, K Kristiansen, S Mandrup, L Grøntved, B Blagoev

**Affiliations:** 1 Department of Biochemistry and Molecular Biology, University of Southern Denmark, Odense M, Denmark; 2 Department of Biology, University of Copenhagen, Copenhagen N, Denmark

## Abstract

The ubiquitin ligase MDM2 is best known for balancing the activity of the tumor suppressor p53. We have previously shown that MDM2 is vital for adipocyte conversion through controlling *Cebpd* expression in a p53-independent manner. Here, we show that the proadipogenic effect of MDM2 relies on activation of the STAT family of transcription factors. Their activation was required for the cAMP-mediated induction of target genes. Interestingly, rather than influencing all cAMP-stimulated genes, inhibition of the kinases directly responsible for STAT activation, namely JAKs, or ablation of MDM2, each resulted in abolished induction of a subset of cAMP-stimulated genes, with *Cebpd* being among the most affected. Moreover, STATs were able to interact with the transcriptional cofactors CRTC2 and CRTC3, hitherto only reported to associate with the cAMP-responsive transcription factor CREB. Last but not least, the binding of CRTC2 to a transcriptional enhancer that interacts with the *Cebpd* promoter was dramatically decreased upon JAK inhibition. Our data reveal the existence of an unusual functional interplay between STATs and CREB at the onset of adipogenesis through shared CRTC cofactors.

Terminal differentiation is the final step in the long sequence of turning a stem cell into a unipotent, specialized cell. With the ongoing obesity epidemic, the adipocyte fate has been attracting more and more attention. These fat-laden cells originate from the mesenchymal stem cells (MSCs), and the understanding of the transcription factor network orchestrating adipogenesis has increased dramatically in the recent years. Within its very core lies the peroxisome-proliferator activated receptor *γ* (PPAR*γ*).^1^ Yet, induction of PPARγ expression does not occur at the onset in the process of turning an MSC into an adipocyte. Its expression is controlled by other proadipogenic transcription factors, of which CCAAT/Enhancer binding protein *β* (C/EBP*β*) and C/EBP*δ* are the best described. Their expression, which is boosted shortly after induction of differentiation, is mainly controlled by members of the signal transducer and activator of transcription (STATs) family as well as cAMP-element binding protein (CREB).^2^

STATs are sequestered in the cytoplasm until activated by tyrosine phosphorylations which leads to translocation to the nucleus and induction of target genes. Of the seven known STATs, only STAT3 and STAT5 are reported to positively affect adipogenesis.^3, 4, 5, 6, 7^ Similarly, CREB has also been shown to enhance adipocyte differentiation.^8, 9, 10^ Interestingly, although a dominant-negative CREB prevents induction of *Cebpb* during differentiation, a constitutive active CREB restores adipogenesis in cells with knockdown of C/EBP*β*, suggesting that CREB facilitates adipose conversion through others means than C/EBP*β*.^8, 10^

CREB is constitutively nuclear and upon phosphorylation recruits general cofactors such as p300/CBP (CREB-binding protein) to activate transcription.^11^ However, CREB recruits more specialized coactivators as well. The CREB-regulated transcriptional coactivators (CRTCs), of which three are known, are only reported to bind and activate CREB.^12^ Interestingly, ectopic expression of a dominant-negative CRTC is able to decrease adipogenesis.^13^

Here we demonstrate that CREB does not have exclusive rights to CRTCs and that it shares these cofactors with the STATs for cooperative regulation of adipocyte differentiation. We show that ablation of the ubiquitin ligase MDM2 leads to dysfunctional STAT activation. Inhibition of the JAKs, which are the main kinases responsible for STAT activation, resulted in decreased binding of CRTC2 to an enhancer region, which interacts with *Cebpd* transcriptional start site (TSS), and dramatically reduced adipocyte conversion.

## Results

### Knockdown of MDM2 prevents *Cebpd* induction and adipocyte differentiation in a p53-independent manner

**Figure 1:**
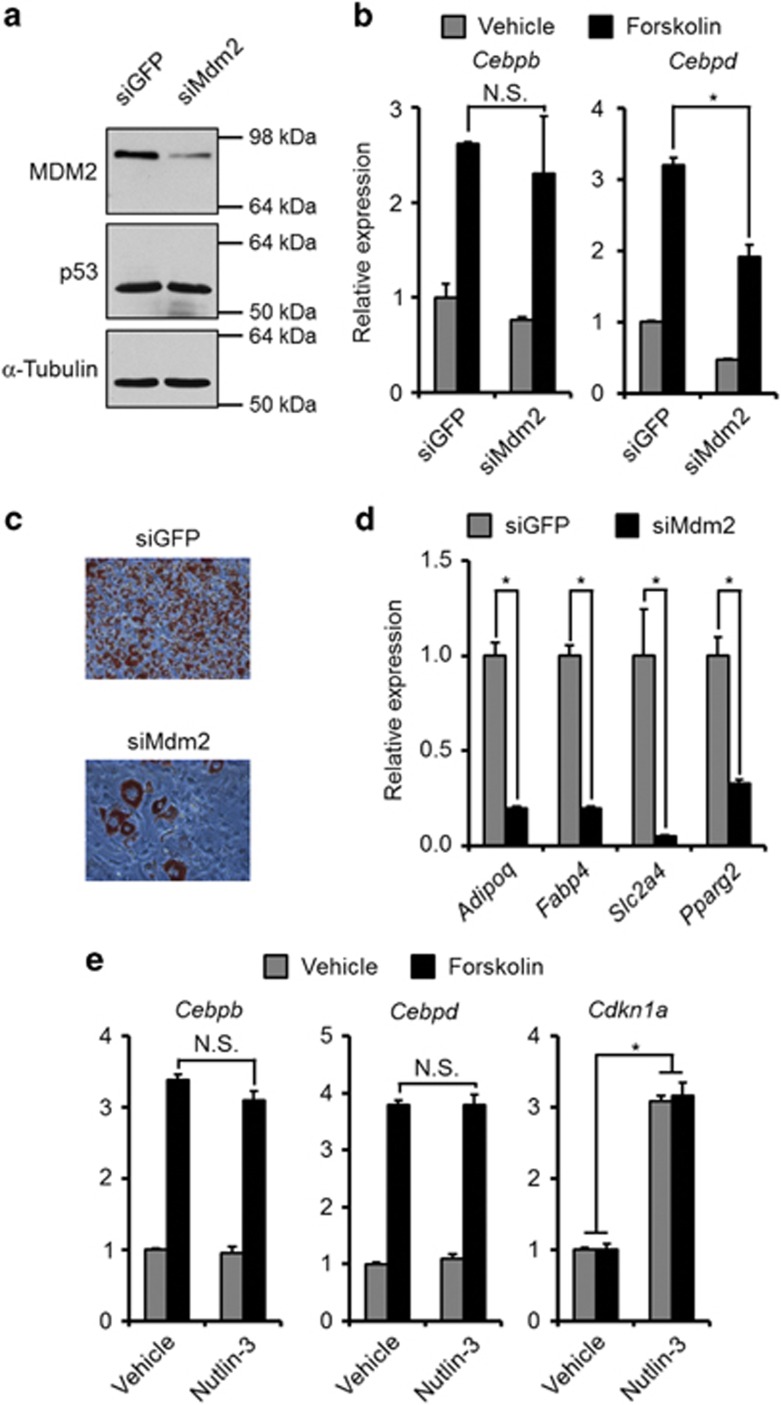
MDM2 knockdown but not Nutlin-3 treatment lowered cAMP-mediated induction of *Cebpd*. (**a**–**d**) MDM2 or control (GFP) was knocked down in confluent 3T3-L1 preadipocytes using siRNA transfection. (**a**) Protein levels of MDM2, p53 and *α*-tubulin 48 h post transfection. (**b**) Transfected cells were stimulated with forskolin or vehicle for 1 h. mRNA levels of *Cebpb* and *Cebpd* were measured by real-time qPCR. (**c** and **d**) Transfected cells were induced to undergo adipocyte differentiation. Degree of adipogenesis was scored by Oil-Red-O staining of triglycerides (**c**) or mRNA levels of adipocyte marker genes by real-time qPCR (**d**). (**e**) 3T3-L1 preadipocytes were treated with either Nutlin-3 or vehicle before stimulation with forskolin. mRNA levels of *Cebpb*, *Cebpd* and *Cdkn1a* were measured by real-time qPCR. *Significance tested using Student's *t*-test, *P*<0.05

In our previous work we have seen a necessity for MDM2 in the induction of *Cebpd*, but not *Cebpb*, at the onset of adipocyte differentiation in mouse embryonic fibroblasts (MEFs). This resulted in a decreased adipose conversion of MEFs lacking MDM2.^13^ We sought to corroborate these findings in a classic preadipocyte cell line. In agreement with the findings in the MEFs, the cAMP-mediated induction of *Cebpd*, but not *Cebpb*, was impaired in 3T3-L1 preadipocytes with knock down of MDM2 (Figures 1a and b). Also, 3T3-L1 cells with decreased levels of MDM2 were less efficient in undergoing adipogenesis (Figures 1c and d).

Despite the importance of MDM2 in regulating p53 abundance, the level of the tumor suppressor was not upregulated in 3T3-L1 cells upon MDM2 knockdown (Figure 1a), indicating that the involvement of MDM2 in regulation of *Cebpd* induction was p53-independent, as reported earlier.^13^ This was further substantiated by the inability of Nutlin-3, which increases p53 levels by preventing its binding to MDM2,^14^ to modulate the cAMP-mediated increase in *Cebpd* expression (Figure 1e). The stimulation of *Cdkn1a* (Cyclin-dependent kinase inhibitor 1a, encoding p21) confirmed that 3T3-L1 cells do possess functional p53 (Figure 1e).

Collectively, these data show the previously reported role of MDM2 in MEF adipogenesis can be recapitulated in the 3T3-L1 preadipocyte model.

### MDM2 deficiency leads to perturbed STAT activation

**Figure 2:**
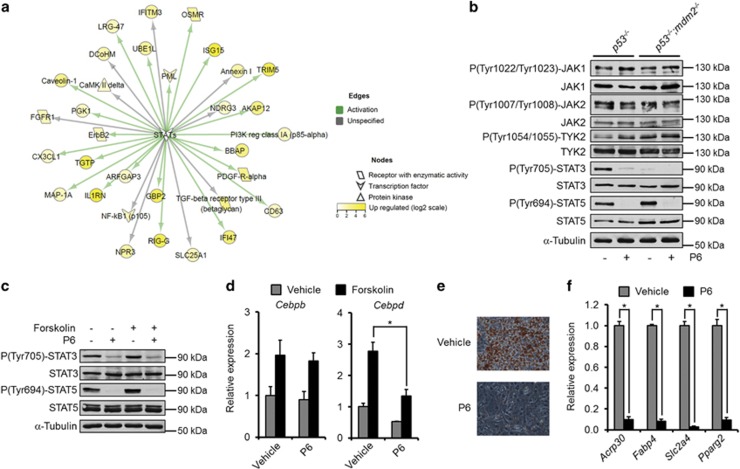
MDM2 is needed for STAT activation. (**a**) Visualization of MS-based SILAC ratios for known STAT targets. Yellow-colored nodes are upregulated in *p53*^*−/−*^ MEFs. (**b**) *p53*^*−/−*^ and *p53*^*−/−*^*;mdm2*^*−/−*^ MEFs were treated with the JAK inhibitor, P6. Protein and phosphorylation levels of JAKs and the adipogenic STATs were measured using western blotting. (**c** and **d**) 3T3-L1 preadipocytes were treated with P6 and/or forskolin. (**c**) Western blot analyses of protein and phosphorylation levels of the proadipogenic STATs. (**d**) mRNA levels of *Cebpb* and *Cebpd* as assessed by real-time qPCR. (**e** and **f**) P6 or vehicle was included during adipogenesis of 3T3-L1 cells. Levels of differentiation were scored by Oil-Red-O staining of triglycerides (**e**) or mRNA levels of adipocyte marker genes by real-time qPCR (**f**). *Significance tested using Student's *t*-test, *P*<0.05

To obtain possible cues for the p53-independent effect of MDM2 on *Cebpd* expression and adipocyte differentiation we used SILAC-based quantitative mass spectrometry (qMS)^15, 16^ to get a snapshot of the global proteome of MEFs with and without *Mdm2*. *p53*^*−/−*^ and *p53*^*−/−*^*;mdm2*^*−/−*^ MEFs were grown in light and heavy stable isotope-labeled amino acids, lysates mixed in equal amounts, digested with trypsin and subjected to qMS (Supplementary Figure S1 and Supplementary Table S3). Interestingly, several STAT targets were higher expressed in cells harboring *Mdm2* (Figure 2a).

Although the protein levels of the two proadipogenic STATs, STAT3 and STAT5, did not differ between MEFs of the two genotypes, the best described activating phosphorylation of STAT3, tyrosine 705, was blunted in cells lacking *Mdm2*. In contrast, the homologous phosphorylation of STAT5 on tyrosine 694 was similar (Figure 2b). These tyrosine residues are mainly phosphorylated by the family of Janus Kinases (JAKs), which is composed of four members: JAK1, JAK2, JAK3 and TYK2. In contrast to the others, JAK3 expression is largely confined to the cells of the hematopoietic system. Curiously, levels of JAK1, JAK2 or TYK2 as well as their activating phosphorylations were identical in cells with and without *Mdm2*. Still, the tyrosine phosphorylations of STAT3 and STAT5 were sensitive to the pan-JAK inhibitor P6 (Figure 2b), suggesting that MDM2 is important in conveying signals from JAKs to at least STAT3.

To explore if inhibition of the JAKs in 3T3-L1 preadipocytes could mimic the result of MDM2 deficiency, we treated these cells with P6. Similar to the situation in the MEFs, the inhibitor prevented tyrosine phosphorylation of STAT3 and STAT5 both in the presence or absence of forskolin in 3T3-L1 cells (Figure 2c). Along the same line, whereas P6 did not affect the cAMP-mediated induction of *Cebpb*, it did impair the ability of the second messenger to augment *Cebpd* expression (Figure 2d). Finally, P6 also prevented adipose conversion of the 3T3-L1 cells (Figures 2e and f).

JAKs have previously been suggested to be necessary for induction of *Cebpb* in 3T3-L1 preadipocytes.^17^ However, the JAK inhibitor used in that study, AG490, is also a potent inhibitor of the epidermal growth factor receptor (EGFR).^18^ We therefore tested if inhibition of members of the EGFR family would affect expression of *Cebpb* and *Cebpd*. Indeed, induction of both transcripts by cAMP was decreased in 3T3-L1 cells treated with the EGFR family inhibitor Canertinib (Supplementary Figure S2A). In agreement with the effect of P6 on *Cebpd* only, two other JAK specific inhibitors, Baritinib and Ruxolitinib, prevented induction of *Cebpd* but not *Cebpb* (Supplementary Figures S2B and C).

Recently, STAT3 was reported to convey signals from TYK2 and thereby regulate brown adipocyte differentiation.^19^ As P6 inhibits the kinase activity of all JAK family members, we included a TYK2-specific inhibitor during forskolin-stimulated and differentiating 3T3-L1 preadipocytes to examine whether TYK2 is responsible for STAT activation in these cells. However, inhibition of TYK2 did neither prevent cAMP-mediated induction of *Cebpd*, STAT3 and STAT5 phosphorylation nor adipocyte differentiation (Supplementary Figure S3).

Collectively, these data argue that MDM2 regulates adipogenesis through the JAK–STAT3–*Cebpd* pathway. To our knowledge, MDM2 has hitherto not been associated with JAK–STAT signaling. However, MDM2 is known to interact with the insulin-like growth factor 1 receptor (IGF-1R), which partially signals through JAKs.^20^ Even though chemical inhibition of the IGF-1R prevented adipogenesis of 3T3-L1 cells, it did not prevent cAMP-mediated induction of *Cebpd*, arguing against an involvement of IGF-1R in the observed MDM2-dependent STAT3 activation (Supplementary Figure S4).

### JAK activity regulates only a subset of cAMP target genes

cAMP is known to regulate the expression of a myriad of genes.^21, 22^ To define the cAMP-activated gene program dependent on the JAKs we performed RNA-seq analyses in both 3T3-L1 cells and the MEFs.

**Figure 3:**
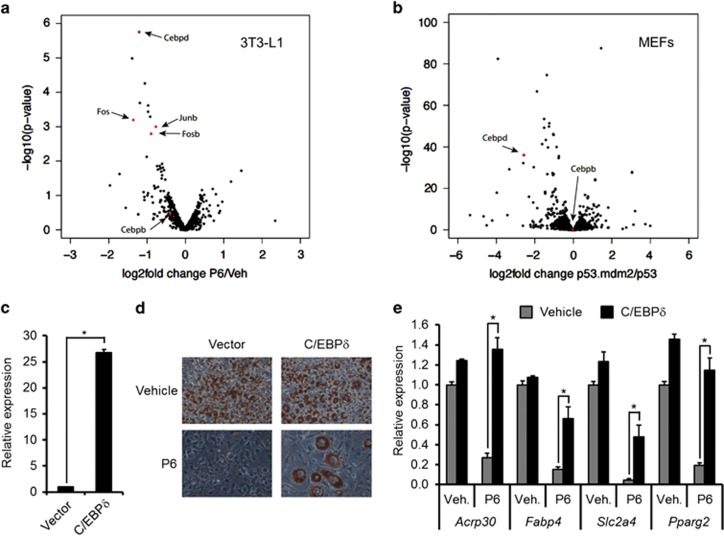
JAK inhibition mainly affects cAMP-mediated induction of Cebpd. (**a**) Volcano plots of the effect of P6 on forskolin-induced genes in 3T3-L1 cells. (**b**) Volcano plots of the effect of *Mdm2* deficiency on forskolin-induced genes in MEFs. (**c–e**) 3T3-L1 preadipocytes were retrovirally transduced with C/EBP*δ* or empty vector. (**c**) mRNA levels of *Cebpd* at day 0 as measured by real-time qPCR. (**d** and **e**) P6 or vehicle were included during adipogenesis of transduced 3T3-L1 cells. Levels of differentiation were scored by Oil-Red-O staining of triglycerides (**d**) or mRNA levels of adipocyte marker genes by real-time qPCR (**e**). *Significance tested using Student's *t*-test, *P*<0.05

First, 3T3-L1 preadipocytes pre-treated with P6 or vehicle were stimulated for 1 h with forskolin or vehicle. As depicted in Figure 3a, only a specific subset of the cAMP-stimulated genes was affected by the inclusion of the JAK inhibitor (the entire list is appended as Supplementary Table S4). Notably, *Cebpd* was the most severely decreased target for P6 in 3T3-L1 preadipocytes, whereas the mRNAs of a few other transcription factors such as *Fos*, *Fosb* and *Junb* were also lowered by the JAK inhibitor.

Second, *p53*^*−/−*^ and *p53*^*−/−*^*;mdm2*^*−/−*^ MEFs were stimulated for 1 h with forskolin or vehicle. The relative differences in cAMP-induction are shown in Figure 3b and Supplementary Table S4. Although more genes were influenced by the lack of *Mdm2* in MEFs compared with P6 treatment in 3T3-L1 cells, *Cebpd* was again among the most severely affected. In agreement with the aforementioned qPCR analyses, *Cebpb* levels were neither influenced by the P6 in 3T3-L1 cells nor by MDM2-ablation in the MEFs (Figures 3a and b).

The global expression analyses in both P6-treated 3T3-L1 cells and *Mdm2*-deficient MEFs strongly suggested that C/EBP*δ* was a key factor in the impaired adipogenesis in the two settings. This notion was further supported by our previous observation that ectopic expression of C/EBPδ was able to restore adipocyte differentiation in *Mdm2*-deficient MEFs.^13^ Consistent with this, forced expression of C/EBPδ was able to at least partially circumvent the P6-imposed block of adipogenesis in 3T3-L1 cells (Figures 3c–e).

Collectively, these data argue that JAK/STAT–MDM2 axis is involved in the regulation of a subgroup of cAMP-regulated genes at the onset of adipocyte differentiation. The adipogenic transcription factor C/EBPδ is one of the genes that is most dependent on this axis. Yet, it is possible that JUN and FOS family members partake in the development as ectopic expression of A-ZIP, which is dominant negative towards C/EBP and JUN families, suffer from a much more severe fat loss compared mice lacking C/EBP*β* and C/EBP*δ*.^23, 24^

### CRTC2 binds to an enhancer near *Cebpd* in a JAK-dependent manner

**Figure 4:**
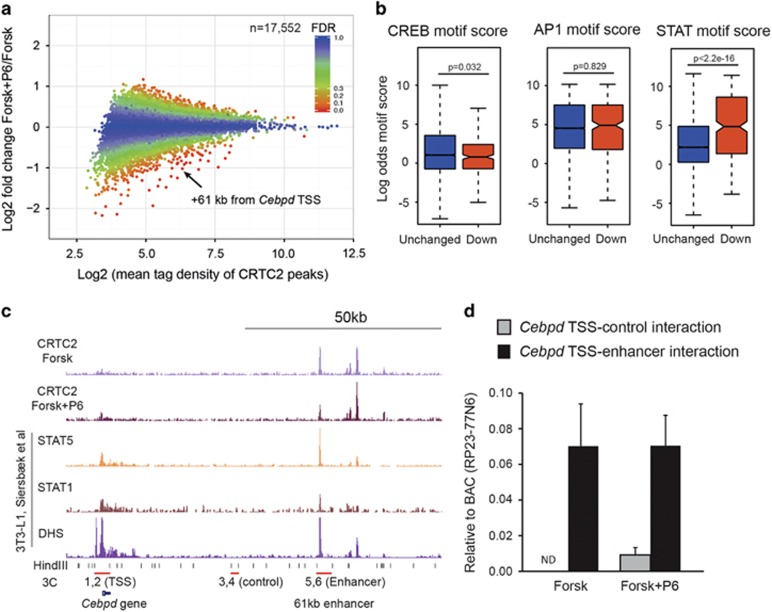
Binding of CRTC2 to *Cebpd*-associated enhancer is hindered by JAK inhibition. (**a–c**) ChIP-Seq of CRTC2 from 3T3-L1 cells treated with either vehicle or P6 was stimulated with forskolin. A total of 17 552 CTRC2 peaks were identified in forskolin-treated cells. (**a**) MA plot showing CRTC binding affected by P6. Differentially bound CRTC2 was identified using EdgeR and the false discovery rate (FDR) of individual CTRC2-binding sites is indicated in the plot. (**b**) Boxplots of CREB, AP-1 and STAT motif strength within CRTC2-binding sites either unaffected or reduced by P6. Unchanged binding is defined as differential binding with FDR>0.8 and reduced binding is defined as differential binding with FDR<0.2 and log2 fold change <0. (**c**) ChIP-seq profiles of *Cebpd* locus. STAT1, STAT3 and DHS data are from Siersbæk *et al.* (REF). Primer sets used for 3C analysis are indicated together with *Hin*dIII sites. Primers 1 and 2 anneal at the *Hin*dIII sites surrounding the *Cebpd* TSS. Primers 3 and 4 anneal at a DNase inaccessible control region 40 kb downstream of the TSS and primers 5 and 6 anneal at the 61 kb enhancer occupied by CRTC2 and STATs. (**d**) 3C analysis of interaction between 61 kb enhancer and control region with *Cebpd* TSS. Ligation efficiency was normalized to ligation efficiency of *Hin*dIII-digested BAC RP23-77N6 containing the *Cebpd* locus

Our previous work suggested an impaired recruitment of the CREB cofactor CRTC2 to the *Cebpd* promoter in *p53*^*−/−*^*;mdm2*^*−/−*^ MEFs upon elevated cAMP levels.^13^ The approach was however biased as we only looked at CRTC2 recruitment to putative CREB-binding sites in the immediate vicinity of the *Cebpd* TSS. To assess possible involvement of CRTC2 in the JAK/STAT–MDM2 axis, we analyzed global CRTC2 chromatin binding in forskolin-stimulated 3T3-L1 cells upon P6 or vehicle treatment. Although CRTC2 was found recruited to more than 17 000 sites in forskolin-stimulated cells, only a minor fraction was significantly affected by JAK inhibition (Figure 4a, 746 sites reduced more than 1.5-fold in response to P6). Motif analyses revealed that STAT-binding sites were more enriched in the pool of sites with reduced CRTC2 binding in the presence of P6 compared with unchanged CRTC2-binding sites. CREB and the closely related AP-1-binding sites were most significantly enriched among both the affected and unaffected peaks (Supplementary Figure S5) and enrichment was similar within affected and unaffected CTRC2-binding sites (Figure 4b).

One of the most JAK-dependent CTRC2-binding sites is located 61 kb downstream of the *Cebpd* gene (Figures 4a and c). Interestingly, this region was previously shown to be bound by members of the STAT family (Figure 4c).^7, 25^ The large distance between the CRTC2-binding site and the *Cebpd* TSS argued that the chromatin had to adopt a configuration that would bring the two regions into spatial vicinity of each other if CRTC2 had to affect transcription of *Cebpd*. Such three-dimensional structural arrangements can be investigated through the chromosome conformation capture (3C) assay.^26^ The schematic of the *Cebpd* locus with the putative CRTC2-bound enhancer, *Hin*dIII digestion sites with their corresponding primers for the 3C analyses, as well as possible structures of the chromatin is depicted in Figure 4c and Supplementary Figure S5B. Analyses of the digestion efficiency of *Hin*dIII near TSS, control and enhancer regions showed that at each site approximately 80% of the chromatin was cut (Supplementary Figure S5C). Interestingly, only when using primers specific for the enhancer-TTS, and not the control-TSS, conformation resulted in amplicon formation, showing that the enhancer region was indeed in close proximity to the *Cebpd* TSS in forskolin-treated 3T3-L1 cells both in the presence and absence of P6 (Figure 4d).

These data suggest that the CRTC2 binding to a STAT site within the +61 kb enhancer near the *Cebpd* promoter is dependent on JAK activity and that this CRTC2-binding site loops and interacts specifically with *Cebpd* TSS.

### CRTC2 and CRTC3 exerts redundant function in the induction of *Cebpd*

**Figure 5:**
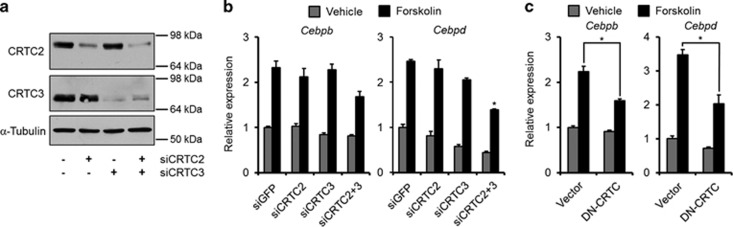
CRTC2 and CRTC3 share function and STAT binding. (**a** and **b**) CRTC2 and/or CRTC3 were knocked down in 3T3-L1 cells. (**a**) Confirmation of knockdowns by western blotting. (**b**) mRNA levels of *Cebpb* and *Cebpd* in forskolin-stimulated 3T3-L1 cells. *Significance tested by one-way ANOVA followed by Tukey's *post hoc* tests, *P*<0.05. (**c**) 3T3-L1 cells were retrovirally transduced with DN-CRTC or empty vector. Forskolin-mediated increase in *Cebpb* and *Cebpd* was analyzed by real-time qPCR. *Significance tested using Student's *t*-test, *P*<0.05

The data shown above argued that CRTC2 is essential for induction of *Cebpd* downstream of cAMP. We therefore examined the ability of 3T3-L1 cells with lowered level of CRTC2 to augment *Cebpd* expression upon cAMP stimulation. However, knockdown of CRTC2 did not prevent the induction of *Cebpd* (Figures 5a and b).

This was seemingly in contrast to the previous observation that ectopic expression of a dominant-negative CRTC (DN-CRTC) was shown to inhibit the cAMP-mediated induction of *Cebpd* in MEFs.^13^ An effect that was repeatable in 3T3-L1 cells (Figure 5c), hinting that another CRTC may be redundant to CTRC2 functions. As expression of CRTC1 is restricted to cells of the central nervous system,^27, 28^ we focused on CRTC3. Interestingly, knockdown of CRTC3 itself did not affect the cAMP-mediated increase in *Cebpd* induction. However, only concomitant knockdown of both CRTC2 and CRTC3 significantly decreased the cAMP-mediated induction of *Cebpd* (Figures 5a and b).

These data show that both CRTC2 and CRTC3 can substitute for each other in the induction of *Cebpd*.

### CRTCs bind STATs and MDM2

**Figure 6:**
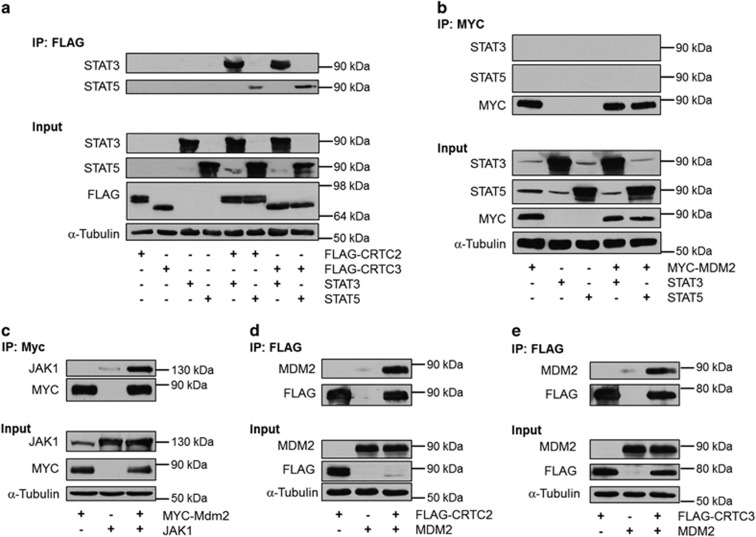
CRTCs can bind STAT3, STAT5 and MDM2. (**a**) 293T cells were transfected with FLAG-tagged CRTCs and/or STATs. FLAG-immunoprecipitates (IPs) were analyzed for STAT3 and STAT5 by western blotting. (**b**) 293T cells were transfected with MYC-tagged MDM2 and/or STATs. MYC-IPs were analyzed for STAT3 and STAT5 by western blotting. (**c**) 293T cells were transfected with MYC-tagged MDM2 and/or JAK1. MYC-IPs were analyzed for JAK1 by western blotting. (**d**) 293T cells were transfected with FLAG-tagged CRTC2 and/or MDM2. FLAG-IPs were analyzed for MDM2 by western blotting. (**e**) 293T cells were transfected with FLAG-tagged CRTC3 and/or MDM2. FLAG-IPs were analyzed for MDM2 by western blotting

In order to understand how the interplay between JAK/STAT, CRTCs and MDM2 could regulate induction of *Cebpd*, we analyzed which of the individual components were able to interact. We found that CRTC2 and CRTC3 were able to bind the two adipogenic STATs, although both CRTCs bound stronger to STAT3 compared with STAT5 (Figure 6a).

MDM2 has previously been shown to interact directly with and modulate the activity of several transcription factors.^29, 30^ It is, however, unlikely that the decreased STAT activity in *p53*^*−/−*^*;mdm2*^*−/−*^ MEFs is due to direct binding of MDM2 to STATs as they failed to interact based on co-immunoprecipitation experiments (Figure 6b). In contrast, MDM2 was able to readily associate with the upstream kinase, JAK1 (Figure 6c). This suggests that MDM2 regulates STAT activation independent of direct binding to the transcription factors. Besides binding to JAK1, MDM2 was also able to interact with both CRTC2 and CRTC3, hinting to a deeper involvement of MDM2 in *Cebpd* induction than just activation of STAT3 (Figures 6d and e).

Collectively, these data show that several of the constituents of the MDM2–JAK/STAT–CRTC network are able to interact.

### Chromatin binding of CRTCs is modulated by P6 and MDM2

**Figure 7:**
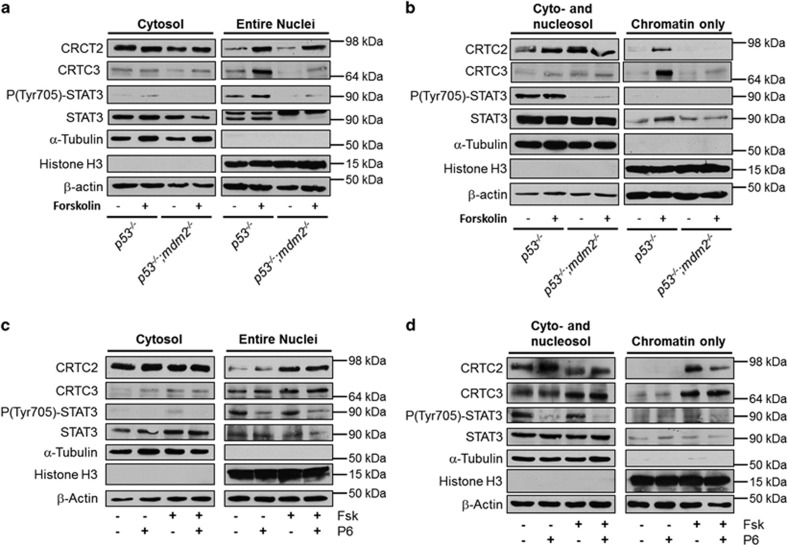
MDM2 regulates localization and chromatin binding of CRTCs. (**a** and **b**) *p53*^*−/−*^ and *p53*^*−/−*^*;mdm2*^*−/−*^ MEFs were stimulated with vehicle or forskolin for 15 min. Cells were then fractioned into cytosolic and nuclei (**a**) or cyto/nucleosol and chromatin (**b**). (**c** and **d**) 3T3-L1 cells treated with vehicle or P6 were stimulated with vehicle or forskolin for 15 min. Cells were then fractioned into cytosolic and nuclei (**c**) or cyto/nucleosol and chromatin (**d**)

Both STATs and CRTCs are cytoplasmic proteins that translocate to the nucleus upon stimulation. We therefore examined the levels of the STAT3 and CRTCs in MEFs separated into cytosolic and nuclear fractions. STAT3 was only found in the nuclei of *p53*^*−/−*^ MEFs (Figure 7a). This correlates with the increased phosphorylation status of STAT3. Interestingly, the two CRTCs had different distributions. Whereas CRTC2 entered the nucleus upon forskolin stimulation in MEFs of both genotypes, CRTC3 translocation was drastically reduced in MEFs lacking *Mdm2* (Figure 7a).

When fractionating cells under more stringent conditions, it is possible to solubilize nuclear proteins together with the cytosol and separate it from the chromatin and large cytoskeletal structures. Interestingly, although CRTC2 was able to enter the nuclei in both *p53*^*−/−*^ and *p53*^*−/−*^*;mdm2*^*−/−*^ MEFs, it was bound less tightly to chromatin in cells lacking MDM2. Consistent with the dysfunctional translocation of CRTC3 in *p53*^*−/−*^*;mdm2*^*−/−*^ MEFs, it was not found in the chromatin fraction of these cells (Figure 7b).

To elucidate if JAK activity was needed for the translocation of the CRTCs, we fractionated 3T3-L1 cells treated with P6 and/or forskolin. Although the translocation of CRTC2 was unaffected by the inhibitor, its binding to chromatin was decreased (Figures 7c and d). On the other hand, binding of CRTC3 to DNA was unaffected by the inhibitor (Figure 7d) again arguing for different modes of regulation of the two related coactivators.

Collectively, these data show that MDM2 regulates the function of CRTC2 and CRTC3 by controlling their chromatin binding and nuclear translocation, respectively.

## Discussion

**Figure 8:**
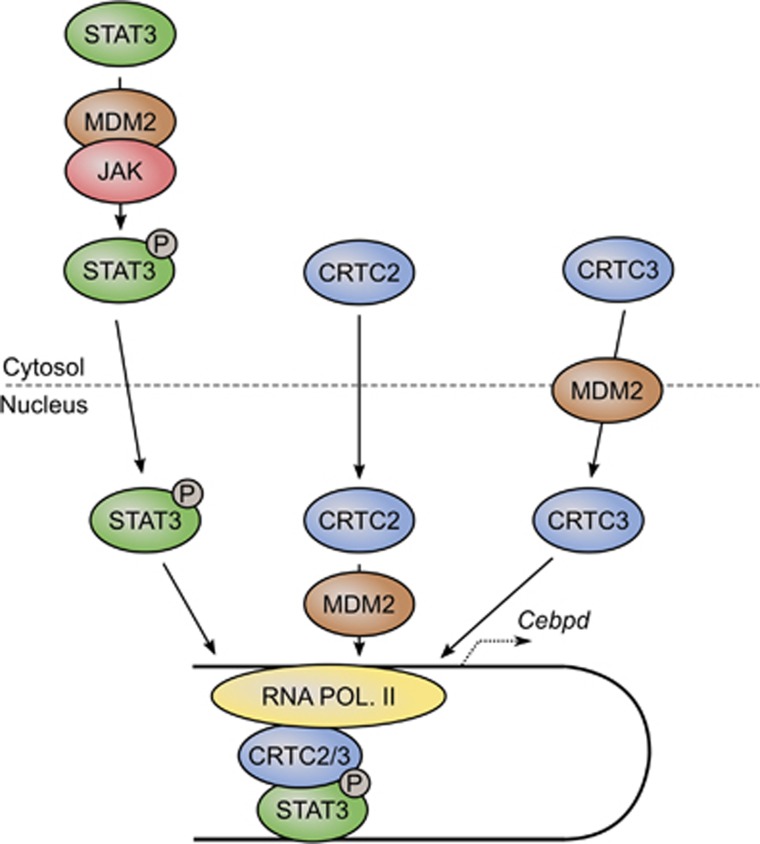
Regulation of STAT–CRTC interplay by MDM2. See text for details

We show here that inhibition of JAKs, the kinases responsible for activation of the STAT transcription factors, attenuated adipogenesis in 3T3-L1 preadipocytes by preventing the cAMP-mediated induction of *Cebpd* but not *Cebpb*. STATs seemingly cooperated with the two cAMP-activated cofactors, CRTC2 and CRTC3, and JAK inhibition also modulated the binding of the CRTCs to chromatin. This interplay was highly dependent on MDM2 as lack of MDM2 caused decreased phosphorylation of STAT3, lowered binding of CRTC2 to chromatin and improper nuclear translocation of CRTC3 upon cAMP stimulation. The suggested interplay is depicted in Figure 8.

It is notable that the STATs were able to bind the CRTC cofactors. Hitherto, these cofactors have to our knowledge only been ascribed to activation of CREB and other members of the basic leucine zipper (bZIP) family of transcription factors.^12, 31, 32, 33^ However, the CRTCs are not the only shared cofactors between bZIPs and STATs. Both families associated with the acetyl transferases p300 and CBP.^34, 35, 36^ As CRTCs in part augmented CREB activity by facilitating the recruitment of p300/CBP to CREB-binding sites,^37^ it is likely that the CRTCs can strengthen the association between STATs and p300/CBP.

Recent insights have indeed shown that transcription factors collaborated in the orchestration of adipogenesis.^2, 25^ Although our results here suggested that the induction of *Cebpd* relied on CTRC–STAT interplay, we strongly believe that the classic partner of the CRTCs, CREB, was also a part of this complex. This hypothesis is supported by the finding that P6 reduced the binding of CRTC2 to CREB-binding motifs (Figure 4b).

CRTC2 and CRTC3 shared overlapping functions in the induction of *Cebpd* by cAMP at the onset of adipogenesis. Interestingly, mice with knockout of either of the two had phenotypical changes in their adipose stores. Adipose mass tended to be lower in mice with ablated CRTC2^33^ and knockdown of CRTC2 decreased the lipid content of differentiating 3T3-L1 cells.^38^ On the other hand, mice lacking CRTC3 displayed a severely decreased adipose mass although this was due to increased energy expenditure in the brown adipose tissue.^28^ Based on the findings presented here, it is, however, possible that a severe defect in adipogenesis *in vivo* would only be evident in mice lacking both CRTC2 and CRTC3 due to redundancy between the two cofactors.

Our data here indicate that MDM2 was vital for conveying the signals from JAKs to at least STAT3, possibly through direct interaction with the JAKs. Besides its kinase domain, the JAKs are composed of an SH2, a pseudokinase and an FERM domain, which all contribute to the regulation of JAK activity.^39^ The FERM domain is of special interest as MDM2 has been shown to bind the FERM regions of the focal adhesion kinase 1 and 2 (FAK and FAK2/PYK2).^40, 41^ Interestingly, the FERM domains of JAKs shared the highest similarities with those of the FAKs compared with FERM domains of other proteins.^42^ For both FAK1 and PYK2, the FERM domain was needed for the positive effect of the kinases on MDM2-facilitated p53 ubiquitination and degradation. It is, however, highly likely that MDM2 returns the favor by regulating activation of FAK/PYK2 as binding partners of the FERM domains controlled the activity of the kinases.^42^

The same is possible for the JAKs. The FERM region has in several incidences been shown to be necessary for receptor binding and JAK activation through conformation changes.^43, 44, 45^ It is therefore likely that by binding to the FERM domain, MDM2 can alter the folding of the JAKs and thereby facilitate their recruitment to receptors and subsequent activation.

Interestingly, whereas CRTC2 was able to shuttle into the nucleus upon cAMP stimulation, CRTC3 failed to do so in the absence of MDM2. However, despite being able to enter the nucleus the binding of CRTC2 to the chromatin was significantly reduced in cells lacking MDM2. These data argue that MDM2 has different modes of regulating CRTC2 and CRTC3. This notion was further strengthened by the observation that overexpression of MDM2 leads to a dramatic downregulation of CRTC2 but not CRTC3, suggesting that MDM2 may ubiquitinate CRTC2 (Figures 6d and e). It is possible that a direct association between MDM2 and CRTC2 as well as ubiquitination of the latter facilitates DNA binding. Ubiquitination comes in many forms and can even promote transcription.^46, 47^ The destructive effect of ectopic expression of MDM2 on CRTC2 could be an artifact from the ectopic expression as MDM2 at low levels leads to monoubiquitination of p53 and only at high levels MDM2 polyubiquitinates p53.^48^

In addition, decreased STAT3 phosphorylation in cells lacking MDM2 probably also contributed to the lowered binding of CRTC2 to DNA, as the JAK inhibitor P6 lowered CRTC2 recruitment to the *Cebpd* enhancer.

## Materials and Methods

### Plasmids

pBABE-puro was a generous gift from Dr Ormond MacDougald. pBABE-puro C/EBPδ was described previously.^13^ pcDNA3 was kindly provided by Dr Jean-Christophe Marine. pCMV FLAG-MYC-CRTC2 was donated by Dr. Jeffrey Meier. pcDNA FLAG CRTC3 was a gift from Dr. Marc Montminy (Addgene, Cambridge, MA, USA; plasmid # 22976).^12^ pRK5 JAK1 was used previously.^49^ pcDNA3 STAT3 was a generous gift from Dr Jim Darnell (Addgene; plasmid #8706).^50^ STAT5 was moved from pMX (kindly provided by Dr Andrew Brooks) to pcDNA3.1.

### Cell culture and differentiation

*p53*^*−/−*^ and *p53*^*−/−*^*;mdm2*^*−/−*^ MEFs were generous gifts from Dr. Guillermina Lozano. All cells were grown in Dulbecco's modified Eagle's medium (DMEM) with 4.5 g/l glucose (Gibco, Thermo Fisher Scientific, Waltham, MA, USA), 100 U/ml Penicillin–Streptomycin (Lonza, Basel, Switzerland) and 2 mM l-glutamin (Lonza). For growth of 3T3-L1, the medium was supplement with 10% calf serum. For growth of MEFs, H4IIE, HEK293T and Phoenix cells as well as differentiation of 3T3-L1 preadipocytes, the medium was supplemented with 10% fetal bovine serum (FBS) (Gibco).

For mass-spectrometry (MS)-based proteomic analysis, MEFs were grown in DMEM deficient in arginine and lysine supplemented with dialyzed FBS (Gibco), 100 U/ml penicillin–streptomycin and 2 mM l-glutamin. Cells were SILAC labeled^15^ with either l-arginine (Arg0) and l-lysine (Lys0) or l-arginine-^13^C_6_ (Arg6) and l-lysine ^2^H_4_ (Lys4) (Cambridge Isotope Laboratories, Inc., Tewksbury, MA, USA).

Stimulation with 10 *μ*M forskolin (LC Laboratories, Woburn, MA, USA) was carried out for 15 min for subsequent protein and ChIP analyses and 1 h for RNA analyses. Vehicle or inhibitors were added to cells 15 min prior to addition of forskolin. Inhibitors used were 500 nM P6 (Pyridone 6) (Calbiochem, Merck, Darmstadt, Germany), 500 nM PPP (Picropodophyllin) (Calbiochem), 10 *μ*M Bayer-18 (Symansis, Timaru, New Zealand), 10 *μ*M Nutlin-3 (Santa Cruz Biotechnologies, Dallas, TX, USA), 10 *μ*M Canertinib (Selleckchem, Houston, TX, USA), 500 nM Baritinib (Sellechchem) or 500 nM Ruxolitinib. All were dissolved in dimethyl sulfoxide (DMSO) (Sigma-Aldrich, St. Louis, MO, USA).

Two days past confluence (denoted day 0), adipogenesis of 3T3-L1 cells was induced by supplementing differentiation medium with 500 *μ*M isobutylmethylxanthine (Sigma-Aldrich), 1 *μ*M dexamethasone (Sigma-Aldrich) and 1 *μ*g/ml insulin (Sigma-Aldrich). From day 2 to day 4, only 1 *μ*g/ml insulin was added to the differentiation medium. Cells were subsequently refed every second day with adipogenic medium.

For Oil-Red-O staining, cells were rinsed in PBS, and fixed for in 3.7% paraformaldehyde for 10 min. After aspiration of the paraformaldehyde solution, cells were rinsed in water, incubated with Oil-Red-O solution (0.3 g Oil-Red-O (Sigma-Aldrich) in 60 : 40 isopropanol /water solution) for 1 h and rinsed several times with water. After the last wash, cells were left with water.

### siRNA-mediated knockdown

RNAiMAX (Thermo Fisher Scientific) was mixed with MISSION esiRNAs (Sigma-Aldrich) in Opti-MEM (Thermo Fisher Scientific) and aliquoted in gelatin-coated plates. Confluent 3T3-L1 preadipocytes were detached by trypsination, suspended in medium, spun down and resuspended in medium. Hereafter, cells were seeded onto esiRNA:RNAiMAX complexes. This was repeated the following day.

### Retroviral transduction

Phoenix cells were transfected with pBABE-based plasmids. At 2 days post transfection, media were isolated, spun down at 1200 *g* for 5 min to remove cellular debris, mixed 1 : 1 with standard media and added to cells. Polybrene (Sigma-Aldrich) was added to a final concentration of 8 μg/ml. The cells were not selected with puromycin.

### RNA purification, reverse transcription and real-time qPCR

RNA was purified using Isol-RNA lysis reagent (5 PRIME, Hilden, Germany) according to the manufacturer's instructions. For generation of cDNA, 1 *μ*g of RNA was reverse transcribed using MMLV (Thermo Fisher Scientific) according to the manufacturer's instructions. Quantitative PCR was performed in 10 *μ*l reactions containing SYBR Green JumpStart Taq ReadyMix (Sigma-Aldrich), 3 *μ*l of diluted cDNA and 300 nM of each primer. Reaction mixtures were preheated at 95 °C for 2 min followed by 40 cycles of melting at 95 °C for 15 s, annealing at 60 °C for 30 s, elongation at 72 °C for 45 s on the LightCycler platform (Roche, Basel, Switzerland). Sequences of primers (Pentabase, Odense, Denmark) sequences are listed in Supplementary Table S1. *Tfiib* mRNA levels were used for normalization.

### Western blotting

Cells were harvested in lysis buffer (50 mM Tris-HCl, pH 8.0, 500 mM NaCl, 1% NP-40, 1 mM EDTA, 0.25% NaDeoxycholate, 1.5% SDS, protease inhibitors (Complete) (Roche), 5 mM NaF, 5 mM *β*-glycerophosphate, 1 mM Na_3_VO_4_) and sonicated 2 × 5 s at 20% amplitude (Q500) (Qsonica, Newtown, CT, USA). For SDS-PAGE, 100 *μ*g of protein were diluted 5 : 6 in Laemmli buffer (0.3 M Tris, pH 6.8, 12% SDS, 40% glycerol, 0.05% bromophenol blue), heated to 95 °C for 5 min and loaded into each lane. After separation, proteins were blotted onto nitrocellulose paper (GE Healthcare, Little Chalfont, UK) using a Hoefer SemiPhor blotter. Membranes were blocked 1 h at room temperature in either 5% milk or 2% BSA in PBS/T (PBS with 0.1% Tween (Sigma-Aldrich)). Incubation with primary antibodies was carried out overnight. Membranes were rinsed with PBS/T, incubated with horseradish-coupled secondary antibodies (GE Healthcare) and rinsed in PBS/T. For detection, membranes were incubated in chemiluminescent HRP substrates (Millipore, Merck, Darmstadt, Germany). Primary antibodies were directed against MDM2 (4B2, a generous gift from Dr. Jean-Christophe Marine), p53 (OP29; Calbiochem), P(Tyr1022/Tyr1023) JAK1 (sc-16773-R, Santa Cruz Biotechnology), JAK1 (J24320, Transduction Laboratories, San Jose, CA, USA), P(Tyr1007/Tyr1008) JAK2 (sc-16566-R; Santa Cruz Biotechnology), JAK2 (06-255; Upstate, Merck), P(Tyr1054/Tyr1055) TYK2 (9321; Cell Signaling, Danvers, MA, USA), TYK2 (sc-169; Santa Cruz Biotechnology), P(Tyr705) STAT3 (sc-8059, Santa Cruz Biotechnology), STAT3 (sc-8019, Santa Cruz Biotechnology), P(Tyr694) STAT5 (4322; Cell Signaling), STAT5 (sc-835; Santa Cruz Biotechnology), CRTC2 (custom-made; Enogene, Nanjing, China), CRTC3 (A302-703; Bethyl Laboratories, Montgomery, TX, USA), P(Ser133) CREB (9198; Cell Signaling), CREB (48H2; Cell Signaling), Histone H3 (9715; Cell Signaling), MYC (sc-40; Santa Cruz Biotechnology), FLAG (F1804; Sigma-Aldrich), *α*-tubulin (T6199; Sigma-Aldrich), *β*-Actin (sc-47778; Santa Cruz Biotechnology).

Where needed, membranes were stripped in 2% SDS, 20 mM *β*-mercaptoethanol, 62.5 Tris-HCl, pH 6.7 for 30 min at 50 °C. Before incubation with antibodies, membranes where blocked in 5% milk or 2% BSA in PBS/T.

### Immunoprecipitation

Cells were rinsed once in ice-cold PBS before addition of lysis buffer (50 mM HEPES, pH 8.0, 150 mM NaCl, 0.5% Triton-X 100, protease inhibitors (Complete; Roche), 5 mM NaF, 5 mM *β*-glycerophosphate, 1 mM Na_3_VO_4_). After 15 min incubation on ice, cells were scraped off and incubated 15 min on ice with a short vortex every 3–5 min. The lysate was spun down at 10 000 × *g* for 10 min and supernatant was precleared with protein A or G beads for 60 min. Precleared lysate was then incubated with anti-FLAG affinity gel (Sigma-Aldrich), anti-MYC affinity matrix (Covance, BioLegend, San Diego, CA, USA) or protein A beads (GE Healthcare) with nonspecific IgG or anti-STAT3 (sc-482; Santa Cruz Biotechnology) for 6 h, and rinsed four times in lysis buffer before SDS-PAGE.

### Fractionation

For separation of cells into cytoplasm and nuclei, cells were rinsed once in ice-cold PBS and scraped off in PBS with 5 mM NaF, 5 mM *β*-glycerophosphate, 1 mM Na_3_VO_4_. After centrifugation (5 min, 700 × *g*, 4 °C) cells were washed once, spun down and resuspended in hypoton lysis buffer (20 mM Tris-HCl, pH 7.5, 20 mM NaCl, 0.2 mM EDTA, protease inhibitors (Complete; Roche), 5 mM NaF, 5 mM *β*-glycerophosphate, 1 mM Na_3_VO_4_). After incubation 10 min on ice, NP-40 was added to a final concentration of 0.02% followed by 10 strokes with a tight piston in a Dounce homogenizer. Nuclei were spun down at 1200 × *g* for 7 min at 4 °C and rinsed twice in hypoton lysis buffer.

For the more stringent fractionation, cells were rinsed once in ice-cold PBS before addition of modified RIP (mRIPA) lysis buffer (50 mM Tris-HCl, pH 8.0, 150 mM NaCl, 1% NP-40, 1 mM EDTA, 0.25 deoxycholate, protease inhibitors (Complete; Roche), 5 mM NaF, 5 mM *β*-glycerophosphate, 1 mM Na_3_VO_4_). After 15 min incubation on ice, cells were scraped off and incubated 15 min on ice with a short vortex every 3–5 min. The lysate was spun down at 10 000 × *g* for 10 min and pellet was rinsed twice in mRIPA.

### Sample preparation and MS analysis

For quantitative comparison of the *p53*^*−/−*^ and *p53*^*−/−*^*;mdm2*^*−/−*^ MEFs proteomes, we applied stable isotope by amino acids in cell culture (SILAC)-based GeLCMS approach essentially as described.^51^ SILAC-labeled lysates from *p53*^*−/−*^ and *p53*^*−/−*^*;mdm2*^*−/−*^ MEFs were mixed 1 : 1, separated on a NuPAGE gel (Invitrogen) and stained with Novex Colloidal Blue (Invitrogen, Thermo Fisher Scientific). After rinsing of the gel, protein bands were excised from the gel, rinsed twice in 50 : 50 EtOH/50 mM ammonium bicarbonate (ABC), dehydrated with acetonitrile (ACN), reduced with 10 mM DTT in 50 mM ABC, dehydrated with ACN, alkylated with 55 mM chloroacetamide, dehydrated with ACN before rehydration in 12.5 ng/ml trypsin in 50 mM ABC and incubated overnight at 37 °C. The following day peptides were extracted by two rounds of 1% trifluoroacetic acid in 30% ACN incubation followed by three rounds of ACN incubation. Acetonitrile was evaporated before desalting peptides on stage tips.

The LC setup was essentially the same as described previously.^52^ The peptide mixtures were analyzed using an Agilent 1100 nanoflow system (Agilent Technologies, Santa Clara, CA, USA) connected online to an LTQ-Orbitrap Velos mass spectrometer (Thermo Fisher Scientific) equipped with a nanoelectrospray ion source (Proxeon Biosystems, Thermo Fisher Scientific). For chromatographic separation, peptides were injected into a fused silica column packed in-house with 3 *μ*m C_18_ beads (Reprosil) (Dr. Maisch, Ammerbuch-Entringen, Germany), applying a 120- min gradient from 8 to 64% acetonitrile in 0.5% acetic acid at a flow rate of 250 nl/min. We set up the instrument methods for the LTQ-Orbitrap Velos in the data-dependent mode and the 10 most intense peaks were chosen for MS/MS fragmentation with CID activation, FTMS resolution was 60 000, normalized collision energy of 35%, activation *q*=0.25 and activation time of 20 ms. Ions selected for fragmentation were dynamically excluded for 45 s, and lock mass ions *m*/*z* 391.284266, 429.0887724, 445.120024, 503.107515 and 519.138815 were used for internal mass calibration.^53^

Processing of the raw files was done with MaxQuant software (version 1.0.13.13) essentially as described.^54^ In brief, peak lists were generated by the Quant console of MaxQuant program with the following parameters used for searching: double SILAC with labels Arg6/Lys4; maximum two missed trypsin cleavages; six most intense peaks per 100 Da interval used for MS/MS peak lists; mass tolerance was 7 ppm on precursors and 0.5 Da (CID) for fragment ions. A fixed modification was carbamidomethyl (C), and variable modifications were oxidation (M) and N-term protein acetylation.

The MaxQuant-generated peak lists were searched by Mascot v.2.3 (http://www.matrixscience.com) against the mouse International Protein Index database v.3.61. The acquired Mascot DAT files together with the raw files were processed and quantified by the Identify console of MaxQuant with the following parameters: peptide, protein and site of modifications false discovery rate was below 1%, as assessed by the number of hits in the reverse database; minimum peptides length was six; and a minimum of one unique peptide for protein identification was required. All unmodified and modified peptides were used for protein quantification based on both razor and unique peptides requiring a minimum ratio count of two. Only the proteins identified by at least two peptides were accepted.

### Bioinformatics

The list of proteins considered to be differing significantly between the two MEF genotypes coming from the SILAC experiment was imported into the MetaCore software (Thomson Reuters, New York, NY, USA). To check how many of them were regulated at transcriptional level by STATs, we used the 'Expand by one interaction' algorithm, centering it in STATs genes and filtering for 'downstream' for direction of the interaction and 'Transcription regulation' for mechanism. From the resulting set, we retrieved the proteins originating from our experiment and used GProX^55^ and Cytoscape^56^ for visualization of results.

### Chromatin immunoprecipitation

Cells were crosslinked in PBS containing 2 mM disuccinimidyl glutarate (ProteoChem, Loves Park, IL, USA) for 30 min, followed by 10 min crosslinking in PBS containing 1% formaldehyde and finally quenched with 0.125 M glycine (all at room temperature). Crosslinked cells were washed repeatedly in PBS, resuspended in lysis buffer (0.1% SDS, 1% Triton-X 100, 150 mM NaCl and 20 mM HEPES pH 7.6) and sonicated using a Bioruptor (Diagenode, Seraing, Belgium). Chromatin was immunoprecipitated using antibody against CRTC2 (A300-637A; Bethyl Laboratories) and Protein A/G agarose beads (Santa Cruz; sc-2003) overnight at 4 °C. IPs were performed in 1 ml aliquots using 3 *μ*g of antibody. Immunocomplexes were washed extensively and chromatin was eluted and decrosslinked overnight at 65 °C. DNA was subsequently phenol/chloroform purified and ethanol precipitated.

### Next generation sequencing

For mRNA-seq 1 *μ*g total RNA was incubated with poly-dT beads to isolate polyadenylated RNA, subjected to fragmentation and cDNA synthesis followed by library preparation (TruSeq RNA Sample Prep kit v2) performed according to the manufacturer's (Illumina, San Diego, CA, USA) instructions. ChIP-seq libraries were constructed according to the manufacturer's instructions (Illumina) as described previously.^57^ Sequencing was carried out on a HiSeq1500 platform (Illumina).

### RNA-seq and ChIP-seq data analysis

Sequencing reads were mapped to the mm9 genome with STAR^58^ and further analyzed using HOMER.^59^ Differential gene expression was determined using DESeq,^60^ based on two independent biological replicates. CTRC2 peaks were identified using HOMER^59^ based on two biological independent CRTC2 ChIP-seq experiments. Replicate concordant CRTC2 peaks identified in forskolin and forskolin+P6-treated cells were used for further analysis. Differential CRTC2 binding was evaluated using EdgeR,^61^ based on two independent biological experiments. The UCSC Genome Browser^62^ was used for data visualization. *De novo* motif analysis was performed using HOMER.^59^ Sequencing data are available from the Gene Expression Omnibus (GEO) under accession number GSE 82282.

### Chromosome conformation capture (3C)

3C was carried out essentially as described elsewhere.^63, 64^ Cells were fixed in 2% formaldehyde for 10 min at RT, quenched with 0.125 M glycine for 5 min at RT followed by 15 min on ice and washed twice with phosphate-buffered saline (PBS). Cells where spun down at 1200 × *g* for 5 min at 4 °C. Approximately 6 × 10^6^ cells were aliquoted and lysed in 10 ml lysis buffer (10 mM Tris-HCl, 10 mM NaCl, 0.2% Igepal CA-420, Complete EDTA-free protease inhibitor cocktail (Roche)), incubated for 30 min rotating in cold room and nuclei was spun down at 1200  × *g* for 5 min at 4 °C. Nuclei was washed once in 1 ml 1.25 × NEBuffer2 (NEB) and resuspended with 1.25 × NEBuffer2 to a total volume of 358 *μ*l. Then 11 *μ*l of 10% SDS was added followed by incubation at 37 °C for 1 h with gently agitation (10–20 r.p.m.). The SDS was quenched by adding 75 *μ*l 10% Triton-X 100 and incubated at 37 °C for 1 h with gentle agitation. Forty microliters were taken out from every aliquot and used as undigested control.

To digest chromatin, 1500U of *Hin*dIII (New England Biolabs, Ipswich, MA, USA) was added to each aliquot and incubated at 37 °C overnight with gentle agitation. Forty microliters were taken out from every aliquot and used as digested control. After digestion, ligation was performed by adding ligation-mixture (1 × ligation buffer (NEB B0202S), 1 mg/ml BSA, 400U T4 DNA ligase (NEB M0202S)) to digested chromatin mixture in a total volume of 1 ml. Ligation was done at 16 °C 4 h-ON followed by 30 min incubation at RT. Reversal of crosslinking and degradation of proteins was done by adding 60 *μ*l 10 mg/ml of proteinase K (03115879001; Roche) to ligated sample and 2,5 *μ*l Proteinase K to digested and undigested controls followed by incubation at 65 °C and shaking at 800 r.p.m. ON. Additional Proteinase K was added (60 *μ*l 10 mg/ml) and incubation at 65 °C was continued for 2 h. Samples were cooled to room temperature and treated with 10 *μ*l (ligated sample) or 0.5 *μ*l (digested and undigested controls) 10 mg/ml RNase A and incubated at 37 °C for 1 h.

DNA was purified by thorough mixing with 1 : 1 phenol pH 8.0 (Sigma P4557) and then spun down at 3500 r.p.m. for 10 min at RT. The aqueous phase was carefully transferred to a new tube. Extraction was repeated using 1 : 1 of phenol pH 8.0/chloroform (Sigma-Adrich). DNA was precipitated by adding 2.5 × volume of ice cold 100% EtOH and 0.1 × volume of 3 M sodium acetate pH 4.5 followed by overnight incubation at −20 °C or at −80 °C for 1 h. Samples were spun down 4 °C for 3 min and washed 3 × in freshly made 70% EtOH. Pellet was resuspended in 25 *μ*l 1 × TE at 37 °C for ~30 min and DNA concentration was determined.

A control library was prepared by digesting and randomly ligating non-crosslinked purified DNA from bacterial artificial chromosome (BAC) (RP23-77N6, 236 kb) (BACPAC Resources, Oakland, CA, USA) that span the genomic region investigated by 3C. BAC RP23-77N6 was digested and ligated essentially as described in Naumova *et al.*^65^ with minor modifications. In short, BAC RP23-77N6 was grown on LB plates containing chloramphenicol and DNA purified using miniprep (Sigma-Adrich) lysis solution followed by addition of 0.7 × isopropanol for precipitation. BAC was digested in a total volume of 420 *μ*l by using 30 *μ*g BAC DNA, 1 × NEBuffer2, 1500 U *Hin*dIII and 1 mg/ml BSA. DNA was purified by very gentle addition of phenol pH 8.0/chloroform followed by very gentle mixing and then spun down at 18 000 × * g* at RT for 5 min. The aqueous phase was transferred to a new tube and precipitated using 0.1 × volume 3 M sodium acetate pH 4.5, 2.5 × volume 100% EtOH and 20 *μ*g glycogen. Digested BAC was ligated in a total volume of 1 ml as described above for 3C sample and purified by adding 1 : 1 volume of phenol pH 8.0/chloroform and precipitated as described above. Pellet was resuspended in 50 *μ*l TE buffer at 37 °C for ~30 min. 3C control library quality was checked by running DNA on 0.8% agarose gel.

Primers (Pentabase) was designed using http://www.pristionchus.org/3CPrimerDesign/ and following the guidelines described in Frohler and Dieterich.^66^ 3C primers are listed in Supplementary Table S2.

Efficacy of primers was tested using twofold dilution series to make sure detection of product was performed within the linear range and test of digest efficiency for all investigated sites (CEBP*δ* TSS, Control and Enhancer) was performed using digested and undigested controls (Supplementary Figure S5C). Finally, detection of interaction was done by real-time qPCR using combination of unidirectional primers (Fw2–Fw4 and Fw2–Fw6 for detection of interaction between *Cebpd* TSS and control sites or enhancer, respectively) and normalized to BAC control library. Standard deviation was taken from two biological replicates and four technical replicates of the 3C protocol in each treatment.

## Abbreviations

3C: chromosome conformation capture
bZIP: basic leucine zipper
C/EBP: CCAAT/Enhancer binding protein
CBP: CREB-binding protein
Cdkn1a: cyclin-dependent kinase inhibitor 1a
CREB: cAMP-element binding protein
CRTC: CREB-regulated transcriptional coactivator
DN-CRTC: dominant-negative CRTC
EGFR: epidermal growth factor receptor
FAK: focal adhesion kinase
IGF-1R: insulin-like growth factor 1 receptor
JAK: Janus kinase
MEF: mouse embryonic fibroblast
MSC: mesenchymal stem cell
PPAR: peroxisome-proliferator activated receptor
qMS: quantitative mass spectrometry
STAT: signal transducer and activator of transcription
TSS: transcriptional start site
