# Supplementary material for: MDM2 facilitates adipocyte differentiation through CRTC-mediated activation of STAT3
Source: Cell Death Dis. 2016 Jun 30;7(6):e2289–. doi: 10.1038/cddis.2016.188 (PMC5108339; doi:10.1038/cddis.2016.188)
Supplement: Supplementary Information [file cddis2016188x1.pdf]

Supplementary items

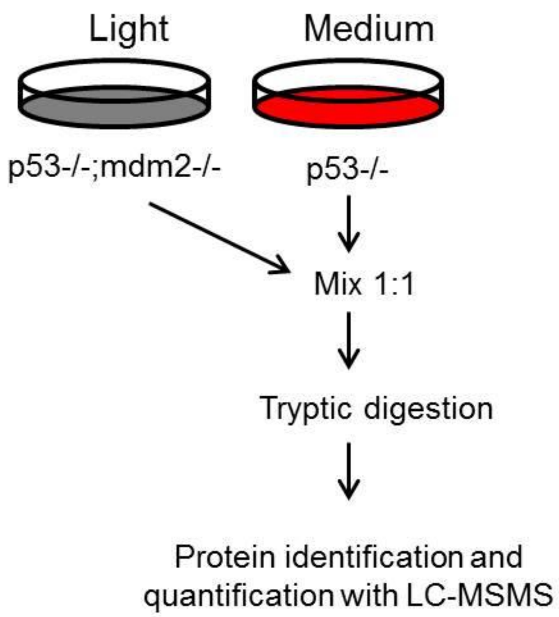

Supplementary figure S1. Workflow for SILAC-labeling and MS analysis of MEFs harboring and lacking *Mdm2*.

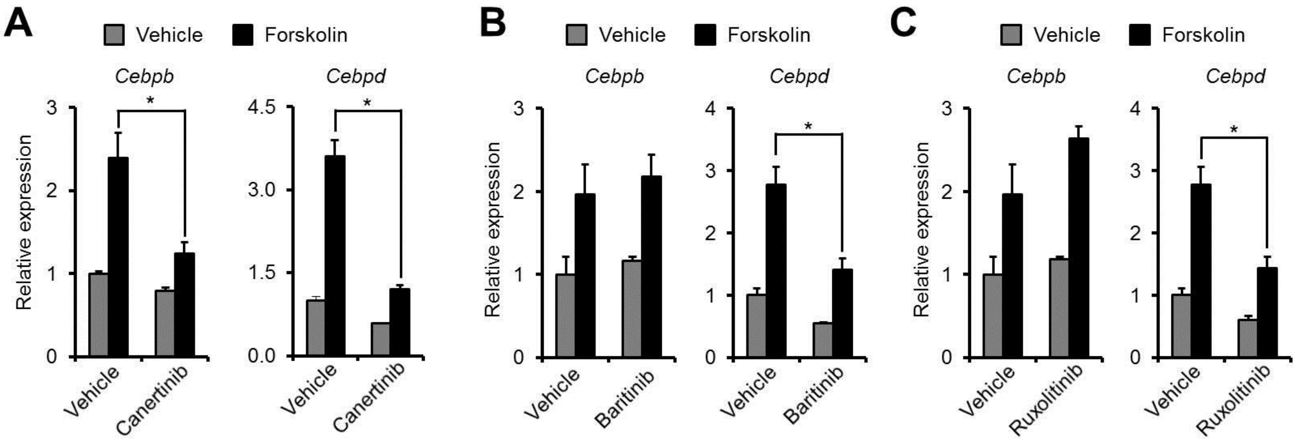

Supplementary figure S2. Inhibition of EGFR prevents induction of both *Cebpb* and *Cebpd*. (A) 3T3-L1 preadipocytes were treated with the EGFR family inhibitor Canertinib and/or forskolin. mRNA levels of *Cebpb* and *Cebpd* as assessed by real-time qPCR. (B) 3T3-L1 preadipocytes were treated with the JAK-specific inhibitor Baritinib and/or forskolin. mRNA levels of *Cebpb* and *Cebpd* as assessed by real-time qPCR. (C) 3T3-L1 preadipocytes were treated with the JAK-specific inhibitor Ruxolitinib and/or forskolin. mRNA levels of *Cebpb* and *Cebpd* as assessed by real-time qPCR. *, significance tested using student's *t*-test, $p < 0.05$.

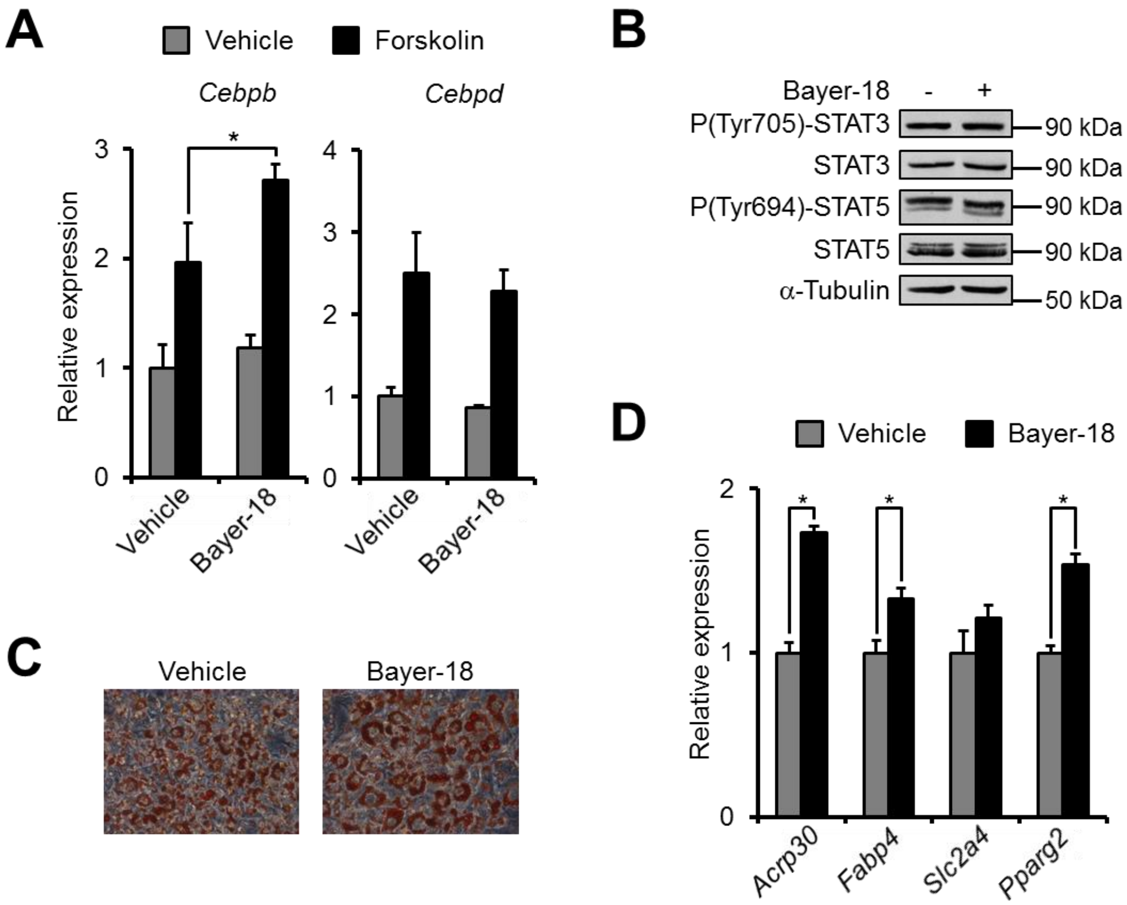

Supplementary figure S3. Inhibition of TYK2 does not prevent adipose conversion of 3T3-L1 cells. (A) 3T3-L1 preadipocytes were treated with the TYK2-specific inhibitor Bayer-18 and/or forskolin. mRNA levels of *Cebpb* and *Cebpd* as assessed by real-time qPCR. (B) Western blot analyses of protein and phosphorylation levels of the proadipogenic STATs upon Bayer-18 treatment. (C+D) Bayer-18 or vehicle were included during adipogenesis of 3T3-L1 cells. Levels of differentiation were scored by Oil-Red-O staining of triglycerides (C) or mRNA levels of adipocyte marker genes by real-time qPCR (D). *, significance tested using student's t-test, $p < 0.05$.

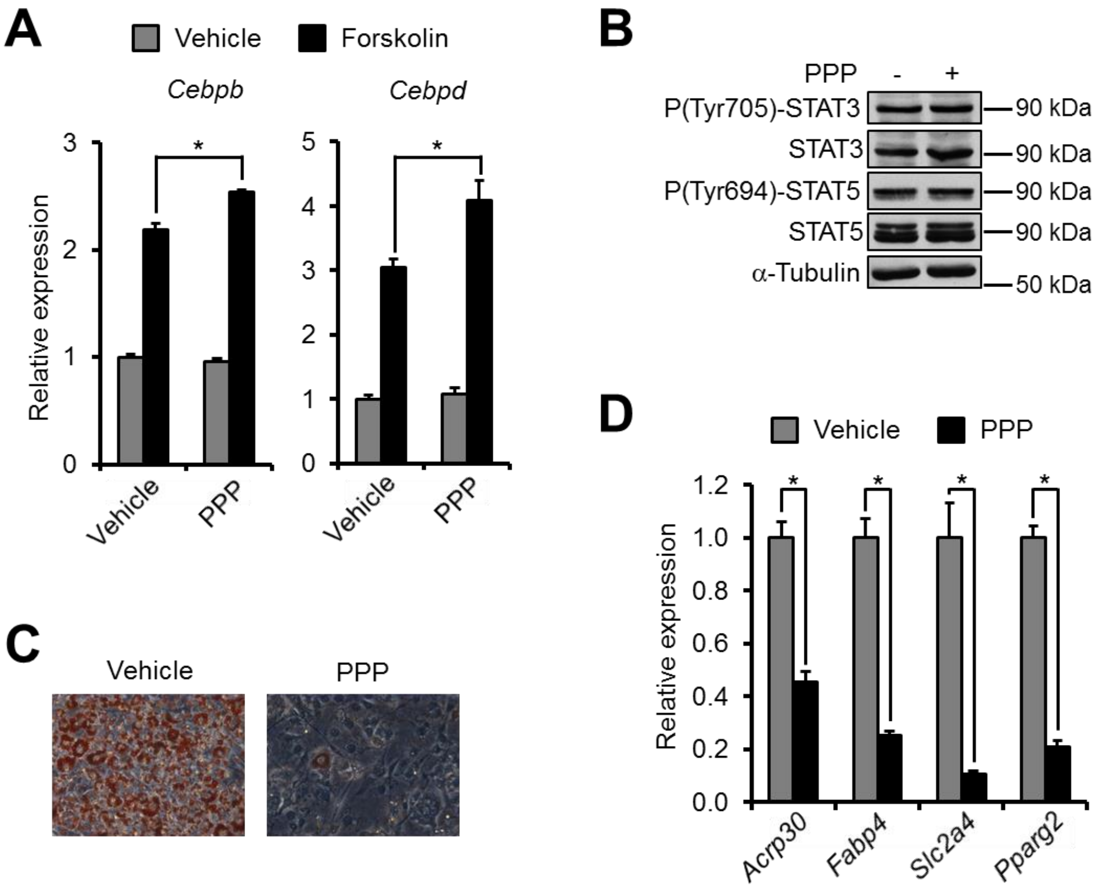

Supplementary figure S4. Inhibition of IGF-1 prevents adipose conversion of 3T3-L1 cells but not induction of *Cebpd*. (A) 3T3-L1 preadipocytes were treated with the IGF-1R-specific inhibitor PPP and/or forskolin. mRNA levels of *Cebpb* and *Cebpd* as assessed by real-time qPCR. (B) Western blot analyses of protein and phosphorylation levels of the proadipogenic STATs upon PPP treatment. (C+D) PPP or vehicle were included during adipogenesis of 3T3-L1 cells. Levels of differentiation were scored by Oil-Red-O staining of triglycerides (C) or mRNA levels of adipocyte marker genes by real-time qPCR (D). *, significance tested using student's *t*-test, $p < 0.05$.

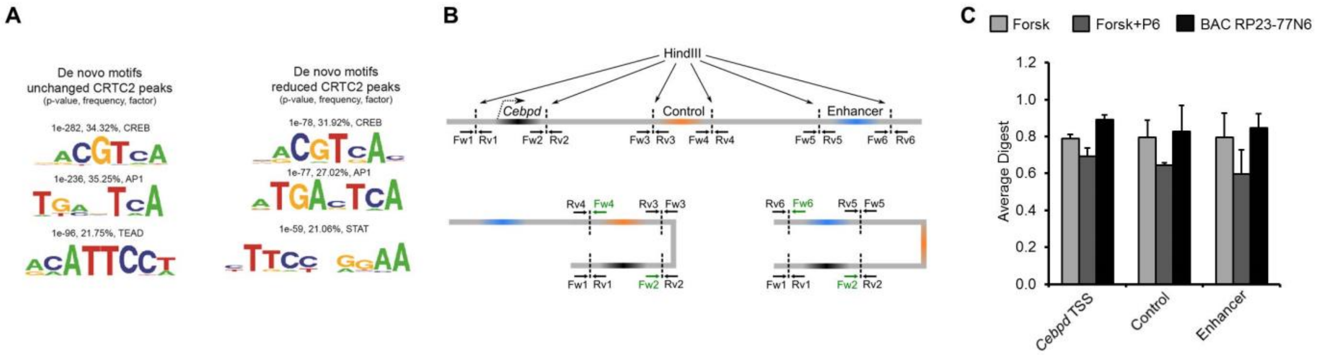

Supplementary figure S5. *Cebpd* promoter binding by CRTC2 and putative folding. (A) *De novo* analysis of CRTC2 binding sites. The three most enriched motifs at either unchanged or reduced by P6 treatment in CRTC2 ChIP-Seq is shown. (B) Schematic of *Cebpd* gene locus with indications of the HindIII digestion sites near the TSS, control and enhancer regions. Primers used for the 3C analysis is indicated. The two tested folding modes of the chromatin are depicted in the lower part. The unidirectional primers labeled in green were used for the 3C analysis. (C) Test of HindIII digestion efficiency in the TSS, control and enhancer regions. Crosslinked chromatin from forskolin and forkolin+P6 treated cells as well as BAC RP23-77N6 is shown.

mRNA	Forward primer	Reverse primer
Cebpb	5’-ggg ttt cgg gac ttg atg c-3’	5’-aca tca aca acc ccg cag g-3’
Cebpd	5’-cca tgt acg acg acg aga gc-3’	5’-tgt ggt tgc tgt tga aga gg-3’
Adipoq	5’-gac acc aaa agg gct cag g-3’	5’-tta gga cca aga aga cct gc-3’
Fabp4	5’-aca gga agg tga aga gca tc-3’	5’-cct ttg gct cat gcc ctt tc-3’
Slc2a4	5’-cag aag gtg att gaa cag agc-3’	5’-ccc tga tgt tag ccc tga g-3’
Pparg2	5’-aca gca aat ctc tgt ttt atg c-3’	5’-tgc tgg aga aat caa ctg tgg-3’
Cdkn1a	5’-cag cga cca tgt cca atc ct-3’	5’-cga aga gac aac ggc aca ctt-3’
Tfiib	5’-tcg acc agc cgt ttg gat gc-3’	5’-tgc tga aag ttc tcc att cag-3’

Supplementary table S1. Sequences for real-time qPCR primers.

Primer name	Primer sequence	Position	Amplicon length
Fw1	AATATGTAGCTCAAGCTGGC	chr16:15,885,084-15,885,257	174 bp
Rv1	GAACAGTTCTCTAGATGGGATACT		
Fw2	CCCAGAACACTAAAACCACA	chr16:15,889,292-15,889,479	188 bp
Rv2	GTGCTTTGTTGTAGCTTTAAAGT		
Fw3	AGGTTTAGACAGAAGGCAAGA	chr16:15,923,466-15,923,630	165 bp
Rv3	CACTAGAAACACATAAAGACATGAGA		
Fw4	GTCCATGGGCTAATGAGAAA	chr16:15,925,422-15,925,582	161 bp
Rv4	TCAATATCCATTCTTACCATTAGGT		
Fw5	AGACAATCTGAATTGCTAGTGATAT	chr16:15,947,487-15,947,659	173 bp
Rv5	AAATAAAACCACAGTAGCTAAGGG		
Fw6	CACTTCCAGGTGCTCTAACA	chr16:15,951,835-15,951,985	151 bp
Rv6	GTGATTCTGGGATCCTGAGAT		
TSS Fw1	TCCCTGTTCCGCCTTTGCTATGTCT	chr16:15,887,108-15,887,246	139 bp
TSS Rv1	CTCCTTGCCTTCCCTCCTTCCTGTT		
TSS Fw2	GCTGCGGAGCCTTGATCC	chr16:15,887,000-15,887,155	156 bp
TSS Rv2	CACTCCTTGCCTTCCCTCC		
Ctrl Fw	TGGGACACTGAAAGGTAGAATCTGTGC	chr16:15,924,396-15,924,569	174 bp
Ctrl Rv	TGTCCACACAACTTAGCAAGGAAACA		
Enhancer Fw1	CATGCACATTTGCCAAGAAGG	chr16:15,948,344-15,948,472	129 bp
Enhancer Rv1	AGTGACCTAGAATAGAACAGC		
Enhancer Fw2	GGGAAACTGCGGACGTGTGTGT	chr16:15,948,410-15,948,580	171 bp
Enhancer Rv2	ACTGCAGCCTCCGACAGTGA		

Supplementary table S2. Sequences for 3C primers
